# Enhancing governance and strengthening advocacy for policy change of large Collective Impact initiatives

**DOI:** 10.1111/mcn.12728

**Published:** 2019-02-22

**Authors:** Isabelle Michaud‐Létourneau, Marion Gayard, Roger Mathisen, Linh Thi Hong Phan, Amy Weissman, David Louis Pelletier

**Affiliations:** ^1^ Department of Social and Preventive Medicine, School of Public Health Université de Montréal Montreal Quebec Canada; ^2^ Department of Family Medicine and Emergency Medicine Université de Sherbrooke Longueuil Quebec Canada; ^3^ Alive & Thrive, Regional Office in Southeast Asia Hanoi Vietnam; ^4^ FHI 360, Asia Pacific Regional Office Bangkok Thailand; ^5^ Division of Nutritional Sciences Cornell University Ithaca New York

**Keywords:** advocacy, Collective Impact, expansion, governance, nutrition, policy change

## Abstract

Nutrition issues are increasingly being addressed through global partnerships and multi‐sectoral initiatives. Ensuring effective governance of these initiatives is instrumental for achieving large‐scale impact. The Collective Impact (CI) approach is an insightful framework that can be used to guide and assess the effectiveness of this governance. Despite the utility and widespread use of this approach, two gaps are identified: a limited understanding of the implications of expansion for an initiative operating under the conditions of CI and a lack of attention to advocacy for policy change in CI initiatives. In this paper, a case study was undertaken in which the CI lens was applied to the advocacy efforts of Alive & Thrive (A&T), UNICEF and partners. The initiative expanded into a regional movement and achieved meaningful policy changes in infant and young child feeding policies in seven countries in Southeast Asia. These efforts are examined in order to address the two gaps identified in the CI approach. The objectives of the paper are (a) to examine the governance of this initiative and the process of expansion from a national to a regional, multilayered initiative, with attention to challenges, adaptations, and key elements, and (b) to compare advocacy in the A&T–UNICEF initiative and in typical CI initiatives and gain insight into how the practice of advocacy for policy change can be strengthened in CI initiatives.

**Key messages**

- Several global initiatives seek to build global partnerships to strengthen the efforts of multiple actors to reach better nutrition outcomes.
- The CI approach can help guide and evaluate the governance of such initiatives.
- The A&T–UNICEF and partners advocacy efforts in seven countries in Southeast Asia met most conditions for an effective CI initiative.
- The backbone of such initiative is the primary element to adapt when expanding.
- Understanding the full policy process, engaging early with policy makers, and using an explicit advocacy approach are some insights that can reinforce the practice of advocacy within CI initiatives.

## 1 INTRODUCTION

In recent years, complex nutrition issues are increasingly being addressed through a variety of global partnerships, platforms, and multi‐sectoral initiatives (Alderman et al., 2013; Levinson, Balarajan, & Marini, 2013). Examples are the Scaling Up Nutrition Movement (scalingupnutrition.org), the Renewed Efforts Against Child Hunger and undernutrition initiative (reachpartnership.org), the Zero Hunger Challenge (un.org/en/zerohunger/challenge.shtml), the Decade of Action on Nutrition (unscn.org/en/topics/un‐decade‐of‐action‐on‐nutrition), and the Global Breastfeeding Collective (unicef.org/breastfeeding). These global initiatives call for the implementation at scale of cost‐effective nutrition‐sensitive interventions (Ruel & Alderman, 2013) and nutrition‐specific interventions (Bhutta et al., 2013). Although they operate in different manners, they all seek to build global partnerships and coalitions to strengthen and align the efforts of multiple actors to reach better nutrition outcomes.

To a significant degree, the success or failure of these initiatives in terms of impact and sustainability depends upon the development of effective governance arrangements (Shiffman et al., 2016). One specific challenge is that many of these initiatives seek to operate at multiple levels (e.g., global, regional, and national), so the optimal governance arrangement at one level may not be optimal at multiple levels. There are a large and increasing number of governance frameworks in the literature that can serve as potential guides (Dodgson, Lee, & Drager, 2002; Gostin & Mok, 2009). There is also rich literature on different modes and forms of governance (Lowndes & Skelcher, 1998; Provan & Kenis, 2008) although this tends to focus more on theoretical concerns rather than practical utility.

The Collective Impact (CI) approach is an example of a practical framework that can guide the governance of such multiorganization initiatives. The CI approach was articulated by Kania and Kramer (2011) in the *Stanford Social Innovation Review*, which became a landmark paper. CI is presented as a high‐performing structured approach for cross‐sector collaboration to achieve large‐scale social impact (Hanleybrown, Kania, & Kramer, 2012). The approach has been used in a broad variety of settings and to address a wide range of problems, from poverty reduction to obesity prevention (Amed et al., 2015; Dipankui, 2017; Flood, Minkler, Hennessey Lavery, Estrada, & Falbe, 2015; Garber & Adams, 2017; Grumbach et al., 2017; Hoey, Colasanti, Pirog, & Shapiro, 2017; Smart, 2017; Thompson & Jocius, 2017). The CI approach can apply to local, national, and regional/global level initiatives (Hanleybrown et al., 2012; Patscheke, Barmettler, Herman, Overdyke, & Pfitzer, 2014). An initiative involving multiple organizations and that uses the CI approach is referred to as a CI initiative. A large effort that involves multiple geographical levels can be called a multilayered CI initiative. There are only a few examples of well‐documented multilayered efforts in the literature on CI that operate explicitly as CI. The Vibrant Communities initiative is one example. Created in 2002, the Vibrant Communities initiative spans over a decade and has been very effective in decreasing poverty in multiple communities across Canada (Gamble, 2010, 2012).

Although the CI approach has received an overwhelmingly positive response, it has also been criticized in academic literature and practitioner forums (Christens & Inzeo, 2015; Flood et al., 2015; Hoey et al., 2017; Le, 2015; Wolff, 2016). One criticism is that the CI approach does not sufficiently focus on advocacy (Flood et al., 2015) and especially not on advocacy for policy change: “[CI] does not include policy change and systems change as essential and intentional outcomes of the partnership's work” (Wolff, 2016, p. 4). This criticism is echoed by other authors (Hoey et al., 2017). Yet a policy change is particularly important for avoiding fragmentation of the work, building the collective efforts of various actors and organizations and addressing issues that cannot be addressed through local action alone (Wolff, 2016). Since its first articulation in 2011, the CI approach has been refined, leading to new generations of CI (Cabaj & Weaver, 2016). The authors of the original CI framework are critics themselves and welcome comments to continue developing it and its implementation.

Given the growing literature and interest across the globe regarding CI, there is a need to examine the governance of broad initiatives through a CI lens, especially those that have explicitly undertaken advocacy for policy change. The advocacy work of Alive & Thrive (A&T), UNICEF and partners is one such initiative. A&T implemented a 9‐year initiative to improve infant and young child feeding (IYCF) policies and practices. During a first phase, in 2012, Vietnam adopted enhanced IYCF policies—maternity leave extended to 6 months and an extended ban on advertising breastmilk substitutes to cover infants up to 24 months—both of which were adopted with a large majority at the National Assembly. The Vietnam experience was well documented, including the steps taken to achieve these policy changes (Hajeebhoy et al., 2013). The Vietnam process, which represents an advocacy approach, has been called the *process for policy change*. It includes four parts: establishing partnerships, building the evidence base, developing messages and materials, and building consensus. In response to growing interest from neighbouring countries, in April 2013, Vietnam organized a 2‐day regional meeting in Hanoi to share their experience and process. This event was the beginning of an expansion from a national experience to a regional movement within Southeast Asia (SEA). Phase 2 of the initiative then began and included the following countries: Cambodia, Indonesia, Lao People's Democratic Republic, Myanmar, Thailand, Vietnam, and Timor‐Leste.

When a CI initiative is successful and expands to become a broader initiative, several adaptations are needed, especially regarding its governance. A number of CI initiatives have experienced and reported this kind of expansion (Gamble, 2010, 2012; Living Cities, 2014a, 2014b). However, while recognizing the need for adaptation, these initiatives appear to be mostly silent on the adjustments required. This point is emphasized in a recent book on CI that calls for more research “to ascertain the overall efficacy of scaling up collective impact approaches” (Ridzi & Doughty, 2017, p. 216). Notwithstanding this, some organizations have proposed diverse strategies to accelerate the growth of CI initiatives, such as the use of regional and statewide intermediaries (Arias, 2014). The need for governance adaptation applies even more when the CI initiative moves from one geographical level to another and becomes a multilayered CI initiative. This is what happened with the A&T–UNICEF initiative. Therefore, the present paper brings insights about the challenges of the expansion process in order to inform and guide future efforts of this type.

Given that the advocacy efforts carried out by A&T, UNICEF and partners in SEA were akin to a multilayered CI initiative that reached significant policy change, this initiative is taken as a case study.

The objectives of this paper are to:

1. examine the governance of this initiative and adaptations that were made to expand from a national CI initiative to a regional multilayered CI initiative and
2. compare advocacy in the A&T–UNICEF initiative and in typical CI initiatives and envision how the practice of advocacy for policy change can be strengthened in CI initiatives.

## 2 METHODOLOGY

### 2.1 Evaluation and inquiry approach

The reflections in this paper were articulated in the context of a real‐time evaluation carried out in seven countries in SEA from May 2015 to March 2017. A research team applied a developmental evaluation (DE) approach to engage with policy advocates throughout the evaluation. Typically, the DE approach supports the development and implementation of an innovation by collecting various types of data that help create feedback to adapt innovations to the emergent and dynamic context (Patton, 2010; Patton, McKegg, & Wehipeihana, 2015). The DE helped track the various activities in the countries over the course of the evaluation.

### 2.2 Case study

This paper presents the results of a rich single‐case study ‐ the A&T–UNICEF initiative ‐ with seven embedded units of analysis ‐ CI initiatives in each country (Yin, 2009). After investigating the progress and experiences in the countries, along with the drivers and triggers of progress (Michaud‐Létourneau, Gayard, & Pelletier, 2019b) and contributions of A&T–UNICEF's approach to progress (Michaud‐Létourneau, Gayard, & Pelletier, 2019a), the activities carried out by the actors were further examined through multiple conceptual lenses to gain additional insights. The CI framework was used to examine the governance of the initiative. The CI framework includes five conditions required to generate effective results: backbone support, common agenda, shared measurement, mutually reinforcing activities, and continuous communication (Table 1, columns 1 and 2).

**Table 1:** Application of the conditions of CI for effective CI initiative

Condition^a^	Definition^a^	Application
Backbone support	An independently funded staff dedicated to the initiative provides ongoing support: guides the initiative's vision and strategy, supports aligned activities, establishes shared measurement practices, builds public will, advances policy, and mobilizes resources.	At the regional level of the multilayered CI initiative, A&T organized large events with UNICEF to build and maintain momentum around IYCF policy enhancement in all the countries. At the country level, A&T strategized with the actors and provided them with capacity building opportunities to advance policy work. They were able to mobilize funding to complement the existing resources and ensure ownership from government and other organizations.
Common agenda	All participants share a vision for change that includes a common understanding of the problem and a joint approach to solving the problem through agreed‐upon actions.	Two elements helped to meet this condition: (a) two regional workshops to foster momentum and define policy asks and (b) an advocacy approach as a joint approach. First, sharing the experience and lessons learned from Vietnam at a 2013 regional meeting was a starting point for a regional initiative. At a 2014 regional workshop, each country team presented their understanding of the problems regarding three policy asks/objectives: the Code of marketing of breastmilk substitutes, maternity protections, and health systems strengthening. Country actors developed road maps with key actions. All country presentations and discussions led to a deep and common understanding of the problems at both levels. Second, a common advocacy approach was a joint approach proposed to improve the situation using the four‐part advocacy process that proved to be effective in Vietnam (Michaud‐Létourneau et al., 2019a).
Mutually reinforcing activities	A diverse set of stakeholders, typically across sectors, coordinate a set of differentiated activities through a mutually reinforcing plan.	A&T–UNICEF actors referred to this strategy as building on comparative advantages, which involved the strategic use of different partners' strengths to build synergy among actions. At the country level, a joint workplan was developed to engage each partner in the collective initiative.
Continuous communication	All players engage in frequent and structured open communication to build trust, assure mutual objectives, and create common motivation.	Communication was initiated at the regional workshops with the various countries. However, it was only over time—and certainly over multiple interactions—that the country actors understood how A&T could support them. Communication over a certain period of time helped to develop the trust and understanding that was necessary for country actors to fully embark on the journey and engage in various actions. Diverse strategies were employed for facilitating communication in‐country. They set up a cloud‐based file sharing system for each country team to facilitate document sharing. The use of new technologies, for example social networking mobile applications, has also been useful to keep in contact with local strategic actors.
Shared measurement	All participating organizations agree on the ways success will be measured and reported, with a short list of common indicators identified and used for learning and improvement.	In the case of the A&T–UNICEF initiative with partners, those measures had not been established at the onset. This is not surprising given that the actors did not commit to use the CI approach and were thus unaware of the five conditions. Rather, they were applying the majority of the conditions of the CI intuitively. At the 2014 Bangkok meeting, the policy objectives were established and agreed upon. These can be considered the ultimate measures of success for this multilayered CI initiative. However, they were insufficient to follow up on the strategic groups' progress. The initiative would benefit in identifying intermediate outcomes that could help to assess progress.

*Note*. CI: Collective Impact; A&T: Alive & Thrive; IYCF: Infant and Young Child Feeding.

a Hanleybrown et al., 2012.

Data collected from the seven countries were examined in relation to these five conditions. Data collection methods included participant observation with A&T staff or representatives in all seven countries, key informant meetings with 98 actors, sporadic in‐depth interviews with 29 actors; reflective practice in which living documents were developed to stimulate reflection with core actors and validate findings, and a desk review of a large number and variety of resources. More information on the data collection can be found in the first paper of this supplement (Michaud‐Létourneau et al., 2019b).

Finally, although the work of multiple actors is acknowledged in this case study, it is important to note that the standpoints and the efforts carried out by A&T may be overly represented compared with the efforts carried out by others, due to the fact that the real‐time evaluation followed primarily A&T actors.

## 3 RESULTS AND INTERPRETATION

### 3.1 Expansion of the initiative into a regional movement and transformation of its governance

This section describes the key elements and challenges involved in expanding a CI initiative into a multilayered one. Table 1 (column 3) provides a description on how the A&T initiative met four of the five conditions of a successful CI initiative, even though it was not formally designed as such.

**Figure 1:**
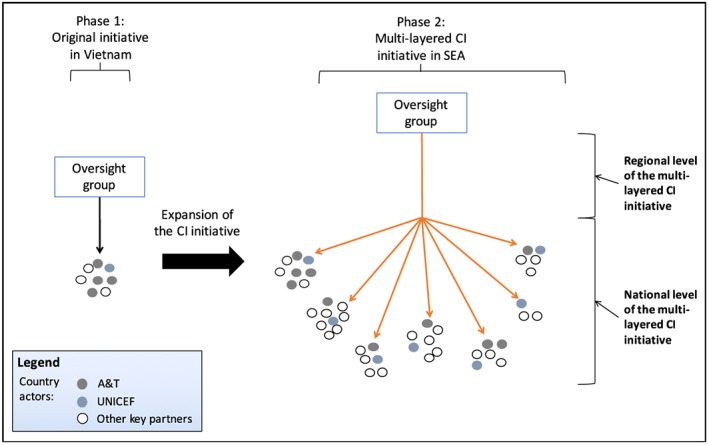
Expansion into a multilayered Collective Impact (CI) initiative. A&T: Alive & Thrive; SEA: Southeast Asia

Typically, in a CI, an oversight group composed of actors from various organizations provides strategic guidance to a backbone that supports the CI initiative. The backbone represents an independently funded staff dedicated to the initiative. The presence of a backbone is the first condition for effective CI initiative, and it helps to put in place the additional four conditions (common agenda, mutually reinforcing activities, continuous communication, and shared measurement). Practically, the backbone assesses and follows up the progress of various working groups. In the original initiative in Vietnam, although we recognize that others led various parts of the work on IYCF policies for their own organization, UNICEF and A&T led the advocacy efforts and therefore represented the oversight group that provided strategic guidance. The role of the backbone was played by A&T, which sought to support the various country actors. At the end of the first phase, A&T and UNICEF began to share their experiences and support other countries in the region. It was the beginning of the expansion to a regional movement, during which the CI became multilayered (see Figure 1). In this new iteration, the first layer of CI involved the country level, that is, the collection of local actors leading the initiative in each country, and the second layer involved the regional level, that is, the collection of seven countries participating to the overall CI.

The expansion into a multilayered CI initiative led to a transformation of the governance of the initiative. A&T SEA and UNICEF EAPRO, 1A&T SEA: A&T Regional Office; UNICEF EAPRO: East Asia and Pacific Regional Office of UNICEF located in Vietnam and Thailand respectively, both continued to guide the overall initiative and played the role of the oversight group, whereas A&T SEA assumed the role of backbone at both levels of the multilayered CI initiative. The remainder of this section highlights three key elements to consider for expansion into a multilayered CI initiative: ensuring the backbone role in‐country, ensuring the use of a joint workplan at the country level, and ensuring communication between the oversight group and country level actors. These elements are based on the experience of A&T, UNICEF and partners during Phase 2 and the related challenges they encountered during the expansion to a multilayered CI initiative. The full list of identified challenges is presented in Table 2.

**Table 2:** List of challenges related to the multilayered nature of the CI initiative

Key elements	Condition of CI	Challenges identified
Ensuring the backbone role in‐country	Backbone support	‐ Limited understanding of A&T strategies and system view by the A&T representatives in‐country ‐ Difficulty of representing two organizations (identity) ‐ Absence of relationship with the government (legitimacy) ‐ Absence of regular contact with local actors
Ensuring the use of a joint workplan at the country level	Mutually reinforcing activities	‐ Limited understanding of the joint workplan by different actors ‐ Turnover of representatives from different organizations ‐ Misunderstanding of the purpose of the joint workplan by partners ‐ Difficulty in generating commitment to the workplan in the presence of multiple competing priorities
Ensuring communication between the oversight group and the country level	Continuous communication	‐ Limited guidance in‐country ‐ Limited bidirectional communication (between the oversight group and A&T representative at the country level)

*Note*. CI: Collective Impact; A&T: Alive & Thrive.

#### 3.1.1 Ensuring the backbone role in‐country

The backbone of a CI initiative bolsters and coordinates the efforts of the actors involved. During the first phase in Vietnam, A&T SEA was based in the country and ensured close support and follow‐up of the different activities. However, when the initiative began to expand, the backbone role evolved, leading to guidance and follow‐up with lower intensity at the country level. First, A&T SEA focal points located in Vietnam travelled periodically to support national actors in other countries, but this was more difficult from outside the countries. This led to another solution, namely, to hire an A&T representative in each country who could ensure this backbone role with closer day‐to‐day follow‐up. This worked well in some cases, but in others, A&T was unable to find the right person to play this role. In such cases, A&T made arrangements for part‐time support from a staff member in another organization already working on related policy issues; we call them A&T representatives.

This evolution of the backbone introduced some challenges. Originally, in Vietnam, a great asset of A&T as a backbone was its flexibility to respond once gaps had been identified. The A&T team had a great understanding of the system and the resources that could be obtained from different organizations. However, after the expansion, when the regional A&T backbone actors were not in the country and instead relied on representatives, the ability to respond was hampered. This was because the country representatives did not have the same system view or knowledge of A&T's budget and budget flexibility and/or they did not have a deep understanding of the A&T advocacy approach. Some representatives also faced an identity challenge because they represented two different organizations in a country, with different mandates. Finally, some representatives missed meetings, events, or updates because of the limited number of days they could devote to doing A&T work.

These challenges affected the progress on IYCF policies by impeding the support that could be provided by the country backbone to the various actors in the countries. Therefore, it remains crucial to ensure that the backbone is able to optimally assume its role at all levels of a multilayered CI initiative. The various ways by which A&T sought to ensure a presence in the countries, despite the absence of an office, illustrate the need for innovative strategies for backbone support. Importantly, the fact that no one country had the same model of representation of the backbone during the entire initiative testifies to the need to reconsider, adapt, and seize opportunities according to the context. Therefore, the regional or higher level entity responsible for a multilayered CI initiative should be prepared to allow and support that kind of flexibility. This emerged as the primary element to consider when envisioning a multilayered CI initiative, as it affects the other two described below.

#### 3.1.2 Ensuring the use of a joint workplan at the country level

CI initiatives build on the work of diverse actors who complement each other by undertaking the activities they are best at, or that best fit within their organizational mandate, to contribute to a collective endeavour. The coordination of different activities involves identifying and supporting mutually reinforcing activities. As presented in Table 1, at a regional workshop in Bangkok in 2014, countries began developing country‐specific roadmaps around three policy objectives for the following years, which led to the creation of a joint workplan. This meeting was itself organized based on the demand for support from a regional meeting in 2013 in Hanoi. The process had started there to frame the overall policy priority setting. Building a joint workplan required the actors to consider other organizations as implementing partners and helped them see how their activities fit into a broader framework. The intent of the joint workplan was not to have organizations commit to new activities, but rather to build on activities that were already planned and that together would increase the likelihood of achieving the desired results.

Partners working closely with A&T on the initiative understood the intent of the joint workplan, but those who were less linked to A&T's activities found this more difficult to understand. Some actors, because they had their own workplan, perceived the joint workplan as an added burden to their ongoing work. Thus, actors had difficulties adjusting their mindset to such a different approach. In addition, a major challenge of the workplan was the turnover of key actors. Those who attended the 2014 Bangkok workshop were not necessarily the same people that the A&T backbone was following up with about the joint workplan progress. This challenge required A&T to repeatedly remind actors where this joint workplan came from and how it was being implemented. A related challenge was the lack of government actors' understanding of the joint workplan, as this approach—not project‐based, but rather technical support to achieve the country's own priorities—differed from many other development projects and initiatives. When these challenges arose, they hampered the CI initiative at the country level, by preventing actors from working closely together. Thus, it remains crucial to ensure that a mindset shift occurs so that the collective can envision how each actor and/or institution can best contribute to the larger plan, beyond the traditional boundaries of institutions. This is an important role for the backbone, but, as indicated, it can be compromised when the country representative/backbone is not fully capable of playing the various backbone roles.

#### 3.1.3 Ensuring communication between the oversight group and the country level

The undertaking of a CI initiative requires frequent and structured communication among the various actors. While working with partners in SEA, A&T at the regional level experienced first‐hand the necessity of maintaining continuous communication with and among many actors in the seven countries. In the original experience in Vietnam, the oversight group, the backbone, and the actors were regularly interacting among themselves, thanks to their close geographic proximity. Thus, communication was less of an issue. However, it was often difficult during expansion. Early in the initiative, some representatives had the feeling that the oversight group at the regional level was not sharing enough information with them. When an A&T representative in a country was asked some questions, this person might not have had all the needed information because they did not receive updates from regional A&T SEA. A&T noticed the communication challenges and initiated regular Skype calls to optimize the follow‐up with the A&T representatives working in the various countries.

### 3.2 Strengthening advocacy for policy change within large CI initiatives

While the first section addressed a gap in understanding the implications of expansion, this section presents five key insights regarding advocacy for policy change within large CI initiatives.

#### 3.2.1 Overcome the fear of engaging in advocacy

Organizations are often reluctant to engage in advocacy due to a misconception of advocacy itself (Almog‐Bar & Schmid, 2014), along with fear of becoming too political (Crutchfield & McLeod‐Grant, 2012). As a result, many non‐profit organizations take the easier option of simply avoiding advocacy (Crutchfield & McLeod‐Grant, 2012). The concern about advocacy may be reinforced by some rules that non‐profit organizations need to abide by. For example, in the United States, foundations need to comply with a broad set of rules (Gates Foundation, 2017), but there is no clear distinction between advocacy for health and social issues and advocacy done by for‐profit organizations. Guidelines on this topic are considered murky and vague (McClung, n.d.). Together, these points highlight an imbalance of power and resources: Corporations strongly advocate for private sector interests and have the funding to hire corporate lobbyists, whereas public health advocates both have an aversion to advocacy and lack sufficient resources to conduct it. Therefore, there is a need to redress this imbalance.

In the specific case of the A&T–UNICEF initiative, advocacy efforts aimed to inform policies that would create an enabling environment for families and fulfil global obligations. These organizations were able to help rebalance the voices of different actors, by supporting the government while making legal expertise available about specific issues (e.g., the Code of marketing of breast‐milk substitutes and maternity protections law) to counteract industry interferences. Indeed, industry often used misinformation and biased arguments to compromise the information that policymakers received about those topics. This readjustment was possible thanks to the long history that UNICEF has with child rights advocacy due to its mission. A UNICEF toolkit is available and describes advocacy as “the deliberate process, based on demonstrated evidence, to directly and indirectly influence decision makers, stakeholders and relevant audiences to support and implement actions that contribute to the fulfillment of children's and women's rights” (UNICEF, 2010). Therefore, UNICEF clearly recognizes advocacy as one of its roles, and the same can be said of A&T. Both organizations were open to publicly mentioning advocacy efforts (which does not mean that they were disclosing publicly all their strategies). In summary, we argue that instead of advocating directly for a certain stance, policy advocates can position themselves as partners and technical experts to inform and assist the policymakers to be in line with international norms and best practices. This distinction may help other organizations that experience a fear of being seen as an opponent of the government.

#### 3.2.2 Build capacity and understanding of the policy process

Actors aspiring to influence policy change need to pay attention to the full policy cycle (including policy development, approval/adoption, preparation for implementation, monitoring and enforcement, and evaluation, learning, and adaptation) rather than adoption alone. In practice, many policy advocates do not possess a good understanding of this overall policy process (Cabaj, 2016), which is a disadvantage when they seek to influence it. This is supported by recent work in a multi‐sectoral initiative to improve nutrition that involved actors from government, non‐governmental organizations, United Nations agencies, and donors. National actors often “lack of an explicit and shared framework for the policy process” (Michaud‐Létourneau & Pelletier, 2017, p. 92). When actors are unaware of the policy process and the full range of stages within the policy cycle, they can have a narrow and fragmented view of what is needed in order to influence any aspect of the policy cycle. In the literature on CI or on the resources made available on the websites of CI initiatives, we could only find a limited number of publications or references and these only dealt with selected aspects of the policy process (Torjman, 2005).

In the A&T–UNICEF initiative, the leaders and some stakeholders in the national strategic groups had a clear understanding of the full policy cycle, from the outset of the initiative. This is not surprising considering the extensive advocacy experience of UNICEF and the expertise developed and learning documented previously by A&T. As a result, during the initiative, they chose to put a strong emphasis on one specific stage of the policy cycle, policy monitoring and enforcement, because that was the stage that appeared the most problematic in many countries. Moreover, they understood that policy change meant more than just having a policy adopted, and they guided the actors accordingly along the whole initiative. We argue that it is important to build capacity of the stakeholders around the whole policy cycle early in the initiative, followed by a more specific emphasis on the most challenging and active stages once the context is better understood and as it evolves over time.

#### 3.2.3 Engage early on with policymakers

An important factor for success is engaging early on in the process for policy change with policymakers, as highlighted by several authors (Brooks, 2017; Kajenthira & Sion, 2017). Many CI initiatives take place at the community level, which causes them to emphasize community engagement over policy advocacy. The emphasis on the community level may also reinforce the notion that advocacy can take place later, once the CI initiative is in a more advanced stage, as suggested in some CI papers (SPARK Policy Institute & ORS/IMPACT, 2018; Torjman, 2005). However, the moment when advocacy takes place should be re‐examined to foster policy change.

In the A&T–UNICEF initiative, which was not a community‐level initiative, actors began to advocate and engage policymakers early in the process and pursued their advocacy efforts during the whole initiative in order to keep the issue on the agenda at all stages of the policy cycle. Thus, there was a great complementarity between policymakers (which represent the government) and other key relevant actors working at the country level. Government allies were able to create the necessary traction in the country, and the other actors (partners) were able to support them in that effort. The synergy emerging from the linkages between those two types of actor is invaluable, each of them bringing different kinds of assets and drawing from a diverse pool of resources in order to facilitate policy change. This suggests that the engagement of key government actors early on in the work can increase the likelihood of a desirable policy change.

#### 3.2.4 Use an advocacy approach explicitly and systematically

The CI framework does not incorporate an explicit and practical way to carry out advocacy for policy change. A simple way to examine advocacy within CI initiatives is to look at the resources that were made available on this topic. We could find some resources on advocacy, such as one developed by the Canadian CED Network (2013). However, the view of advocacy often seems limited to the development of a campaign, even sometimes represented as the only way to reach policymakers. In a comprehensive study that investigated multiple CI initiatives (SPARK Policy Institute & ORS/IMPACT, 2018), although some initiatives have reached policy change, the document does not provide extensive details on the advocacy strategies or approaches that contributed to successful policy change. Although a few strategies were documented, those appeared sparse and did not seem to follow a detailed advocacy approach. At the other end, the CI initiatives that did not appear to have policy advocacy strategies were perceived as less successful in terms of policy and systems change (SPARK Policy Institute & ORS/IMPACT, 2018). Some CI leaders and CI initiatives may use advocacy strategies based either on their experiences or on their intuition, but strengthening the CI framework with the inclusion of an explicit advocacy approach appears to be imperative in order to provide more guidance.

**Figure 2:**
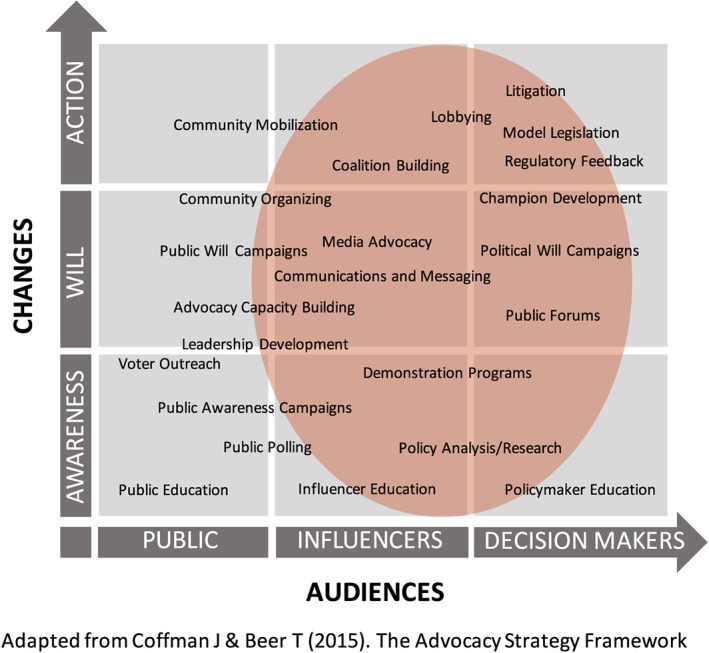
Mapping of the Alive & Thrive (A&T)–UNICEF advocacy efforts on the Advocacy Strategy Framework

For the A&T–UNICEF initiative, there was an explicit advocacy approach at the heart of the work. This advocacy approach contributed to advances within the policy cycle as demonstrated in a companion paper (Michaud‐Létourneau et al., 2019a). Although it may appear simplistic to propose an advocacy approach to undertake advocacy, the four‐part process used by the actors has been very effective in guiding the development of advocacy strategies responding to the context. A mapping tool called the Advocacy Strategy Framework (Coffman & Beer, 2015) 2This tool is offered as an alternative way for articulating theory of change for advocacy initiative to the more conventional linear way. can be used to examine more specifically the advocacy strategies used in the A&T–UNICEF initiative with partners, as illustrated in Figure 2. The figure shows, with a circular shadow, the space of the A&T–UNICEF initiative: it primarily targeted influencers and decision makers by seeking to foster changes along the continuum on the Y‐axis (awareness, will, and action). On the basis of these insights, we argue that the use of an explicit advocacy approach that targets such a broad space (seeking multiple changes of a diversified audience) could increase the likelihood of achieving policy change for CI initiatives. Two practical, simple, and illustrative guides on the advocacy approach used ‐ the four‐part process (A&T, 2016) and on the tool for mapping advocacy strategies (Coffman & Beer, 2015) can be found online. Those could help practitioners working within CI initiatives to develop and classify their advocacy strategies and tactics into an explicit advocacy approach.

#### 3.2.5 Support evidence generation and use data strategically

In the CI approach, using data appears to lie under the responsibility of the backbone. A core document on the backbone of the CI presents three specific roles for staff from the backbone: executive director, facilitator, and data manager. The functions of executive director include “Advance policy: advocate for an aligned policy agenda; and stay on top of policy developments that impact the effort” (FSG & Collective Impact Forum, 2014). In this publication, the function of the backbone regarding advocacy appears underdeveloped, without providing any detail or linkages to the policymaking process besides staying informed. In addition, this specific backbone function does not appear to be connected to data, which misses the importance of data and evidence for advocacy. On the other hand, the data manager role seems only linked to “establish shared measurement practices” and to “support aligned activities.” The CI approach discusses several ways of using data, but those do not seem to be closely linked to advocacy. Instead, data are used to communicate to a partner, for example, why they should get involved in a CI initiative (Raderstrong, 2015) or data are used to understand and assess progress (Living Cities, 2016; Raderstrong & Nazaire, 2017).

A major difference in the A&T–UNICEF initiative is that, for the backbone, being a data manager also implied helping to develop the evidence base, materials, and messages for advocacy. The generation of evidence was strategically planned to provide pieces of information that would help policymakers make informed decisions for different policy issues. For example, in some countries, policymakers wanted to know how much would the implementation of a new policy on maternity protection cost to the government. With the assistance of key actors, the backbone generated evidence on the costs under various scenarios, including the costs of not breastfeeding (Walters et al., 2016), which made a strong argument for policymakers on the importance of protecting breastfeeding. Table 3 provides an illustration of the forms of evidence generated and how it was used strategically for advocacy purpose. This table complements Figure 2 by showing how different forms of evidence were assembled to create sophisticated advocacy strategies and influence various audiences. Of note is that A&T was skillful at finding different organizations willing to share resources and fund studies, rather than always funding the studies themselves. This suggests advocacy need not require extensive funding, if the backbone is skilled at strategically using existing data and facilitating a process in which many organizations could pool their resources for evidence‐based policy change by decision makers.

**Table 3:** Illustration of forms of evidence generated and used strategically in advocacy efforts

1. Evidence generation			2. Strategic use of data			
Forms of evidence	Description	Example (products or means)	Strategy/use	Objective	User	Target audience
Economic	Studies that allow to attribute a cost to certain behaviours (e.g., breastfeeding or not breastfeeding) or health outcomes	Cost of not breastfeeding	Launch of the *Lancet* series	‐ Attract high‐level attention ‐ Convince policymakers who manage budget	Group of strategic actors	High‐level actors Policymakers Potential champions
Empirical	Pilot projects that demonstrate positive results and show the “how‐to” (proof of concept)	Workplace lactation programme (demonstration project)	‐ Inauguration event ‐ Tool kit ‐ Presentation in different forums	‐ Showcase that a certain course of action is possible ‐ Attract high‐level attention ‐ Incentivize companies to apply the same model	Group of strategic actors	Companies Ministries
Sociocultural	Studies that investigate the perspectives of different individuals within society and how factors affect their behaviours	Opinion leader research	Small group discussion	‐ Identify entry point or frames for arguments ‐ Discuss with strategic actors to tailor subsequent actions	Backbone	Group of strategic actors
Legal	Information on the legal frameworks or law‐making process	Legal reviews (retrospective or prospective)	Small group discussion	‐ Understand the law‐making process ‐ Identify entry points	Backbone	Group of strategic actors
International	Reference to international conventions or documents	‐ World Health Assembly resolutions ‐ Convention on the rights of the child	Opinion editorials (Op‐Eds) Letters	‐ Provide the arguments from recognized institutions ‐ Incentivize due to an international source	Group of strategic actors	Government
Scientific or technical	Technical information that are typically found in journal article or academia and come from studies done with solid design	‐ Efficacy or effectiveness of various interventions ‐ Formative research carried out in local setting	Presentations in different forms	‐ Provide evidence‐based findings for diverse audiences ‐ Evidence can be tested in different settings	Backbone Group of strategic actors	Group of strategic actors Government

## 4 CONCLUSION

This paper provides insights about two issues that are important in CI initiatives but have not been extensively studied in earlier literature: expansion into a multilayered CI initiative and use of policy advocacy in CI initiatives.

With regard to expansion, a transformation of the governance structure seems required to ensure that the regional or higher level backbone can effectively ensure its role at all levels and follow‐up and support the actors in‐country. In addition, it is crucial to ensure that a mindset shift occurs among country actors so they understand the intent of a joint workplan. Finally, communication with and among actors is of major importance but presents special difficulties because of geographic and organizational distance, requiring strong mechanisms to be put in place.

With respect to policy advocacy, this experience highlights five significant elements to reinforce its practice for initiatives operating under the conditions of CI. First, it is necessary to recognize and overcome the reluctance or fear of engaging in advocacy. Second, there is a need to strengthen capacity for advocacy, including an appreciation of the full range of stages in the policy cycle and what strategies are needed at each stage. Such strategies are described in more detail in a companion paper (Michaud‐Létourneau et al., 2019b). Third, there is a need to engage early on with policymakers (legislators and/or government allies). Fourth, the use of a clearly defined and explicit advocacy approach is fundamental to success if the initiative seeks to achieve policy change. And fifth, evidence generation and the strategic use of data, both fostered by the backbone, are crucial for advocacy and need not be resource constrained.

Finally, it bears emphasizing that the development of partnerships among A&T, UNICEF, and other organizations was the first element of the four‐part advocacy strategy. This was amply demonstrated in the authentic interpersonal relationships, trust, and respect seen among various actors. This represents an important element that warrants explicit attention in the many (global) initiatives seeking to address complex problems through collaboration. Therefore, initiatives following a CI approach should consider reinforcing the advocacy component, as it can strongly bolster the partnership needed to achieve systems and policy change.

## ETHICS APPROVAL

Ethics committees at both the University of Sherbrooke and FHI 360 approved the research protocol.

## CONFLICTS OF INTEREST

The authors declare that they have no conflicts of interest.

## CONTRIBUTIONS

IML conceptualized this real‐time evaluation and played a leadership role at all stages with DLP providing advice throughout. IML and MG conducted data analysis, interpretation of results, and drafting of the different sections of the manuscript. IML was the main data collector, with participation from DLP, RM, PTHL, and AW depending of countries. IML and MG drafted the manuscript, and the other authors all commented the various sections. All authors read, commented, and approved the final manuscript.

## FUNDING

Alive & Thrive is funded by the Bill & Melinda Gates Foundation, the governments of Canada and Ireland and is managed by FHI 360.
